# A Narrative Review of the Assessment and Treatment of Physical Impairments Commonly Seen in Sarcoma Cancer Survivors Using a Rehabilitative Approach

**DOI:** 10.3390/cancers17010006

**Published:** 2024-12-24

**Authors:** Adrian Cristian, Nandita Keole, Romer Orada, Jayhyun Seo, Austin Guerrina, Arun Maharaj

**Affiliations:** 1 Cancer Rehabilitation, Miami Cancer Institute, Baptist Health South Florida, Miami, FL 33176, USA; romero@baptisthealth.net; 2 Department of Physical Medicine and Rehabilitation, Mayo Clinic Hospital, Phoenix, AZ 85054, USA; keole.nandita@mayo.edu; 3 Rush Medical College, Chicago, IL 60612, USA; jayhyun_seo@rush.edu; 4 Herbert Wertheim College of Medicine, Florida International University, Miami, FL 33199, USA; aguer244@med.fiu.edu; 5 Miami Cancer Institute, Baptist Health South Florida, Miami, FL 33176, USA; arun.maharaj@baptisthealth.net

**Keywords:** sarcoma, rehabilitation, cancer survivorship, quality of life

## Abstract

Background: Sarcomas are a rare, complex group of malignancies characterized by numerous clinical and pathological features. Sarcomas originate from various tissues such as fat, muscle, bone, nerves, blood vessels and connective tissues. Typically, the treatment of sarcomas consists of surgery, chemotherapy, and radiation therapy. The location, type, stage, and treatment of sarcomas contribute to the development of physical impairments affecting joint and limb function, peripheral nerves, strength, and mobility, diminishing physical function and quality of life. Traditionally, rehabilitative therapy has been implemented after cancer treatment to help improve physical impairments imparted from cancer therapy. Recent work in rehabilitative medicine has explored the implementation of rehabilitative therapy prior to beginning cancer treatment (pre-habilitation) to help mitigate the detrimental physical consequences seen in sarcoma patients. Objective: The objective of this narrative review is to examine the physical impairments affecting sarcoma patients after cancer therapy and the impact of rehabilitative medicine on physical function and quality of life when implemented before and after cancer treatment.

## Simple Summary

Sarcomas are rare types of cancer that originate from various tissues such as fat, muscle, bone, nerves, blood vessels and connective tissues. Typically, the treatment of sarcomas consists of surgery, chemotherapy, and radiation therapy. The location, type, stage, and treatment of sarcomas contribute to the development of physical impairments affecting joint and limb function, peripheral nerves, strength, and mobility, diminishing physical function and quality of life. Traditionally, rehabilitative therapy has been implemented after cancer treatment to help improve physical impairments imparted from cancer therapy. This review article aims to examine the physical impairments commonly seen in patients living with sarcoma during and after cancer therapy and their treatment utilizing rehabilitative interventions to improve the physical function and quality of life of those affected by it.

## 1. Introduction

Sarcomas are a conglomeration of rare malignancies with over fifty distinct histological subtypes. Sarcomas are characterized by a wide range of clinical and pathological features, originating from various tissues such as fat, muscle, nerves, blood vessels, bone and connective tissue. Sarcomas are divided into two main subtypes: soft tissue sarcomas (STS) and bone [1] sarcomas. STS originate in muscle, fat, tendons, nerves, blood vessels, and lymphatic tissue, while bone sarcomas are rarer and originate in bone or cartilage [2]. Due to the uncommon, aggressive nature of sarcomas as well as the primary location of origin (muscle and bone), physical impairments not only manifest before treatment, but treatment-related disablement reduces mobility and quality of life post-treatment into survivorship [3,4]. Thus, there is a growing focus on researching the most effective rehabilitative medicine in sarcoma patients before (pre-habilitation) and after therapy to help mitigate treatment-related physical consequences with the end goal of improving mobility, independence, and quality of life.

With any form of cancer, there is a wide spectrum of symptom burden that impacts a patient’s life. Sarcoma patients experience some of the more physically limiting symptom burdens such as extreme muscle or bone pain, unexplained fractures as well as fatigue and insomnia [1,5]. Sarcomas are often difficult to diagnose. Immediate referral to a specialist is advised if a patient with soft tissue mass experience increased size of mass and/or deep fascial pain or, in the case of bone sarcoma, if there is severe, persistent bone pain or an unexplained fracture [6]. Thus, limited mobility and physical impairment in sarcoma patients manifest well before treatment is implemented. Post-treatment physical impairment adds to the complexity of rehabilitative therapy for sarcoma patients. Pre-habilitation of sarcoma patients prepares them for withstanding treatment-related toxicity and improving rehabilitation post-treatment; however, delay in seeing a patient with sarcoma by a rehabilitation specialist is a missed opportunity that can limit the benefits of this intervention [7,8,9].

This narrative review will provide a brief overview of the epidemiology, diagnosis, treatment of sarcomas followed by an assessment and treatment of physical impairments before and at diagnosis, after cancer therapy, and the impact of rehabilitative medicine on physical function and quality of life when implemented before and during therapy and into survivorship.

Literature Search Strategy: A literature review was conducted using the following PubMed medical subject heading (MeSH) terms: “soft tissue sarcoma clinical practice”,” sarcoma/rehabilitation”, “hemipelvectomy, “upper limb,” post amputation” “lymphedema”, “physical therapy modalities”, “exercise therapy”, “prehabilitation”, “physical impairments”, “gait disorders”, “balance”, “radiation injuries”, “radiation fibrosis”, “cancer survivors”, “quality of life”, “activities of daily living”, “physical impairments”, “motor activity”, “cancer survivors” for the time period 2014–2024 in adults 19 years or older.

## 2. Epidemiology, Diagnosis and Treatment

STS account for approximately 1% of adult cancers, and two-thirds originate in the lower extremities [10,11]. Several risk factors have been identified for the development of STS, including prior local tissue injury from surgery, radiation therapy, burns, as well as exposure to chemicals like vinyl chloride and Agent Orange. Immunocompromised conditions and rare genetic syndromes, such as Li-Fraumeni Syndrome and Neurofibromatosis Type 1, are also associated with increased risk of sarcoma development [11]. Because of the rarity and need for multimodal treatment, management of sarcomas should ideally be conducted in a sarcoma center, since the expertise of surgical sub-specialists has been shown to improve clinical outcomes [12]. Current treatment goals are to improve survival, prevent recurrence, and mitigate chance of metastasis while simultaneously minimizing impairments and disability. Treatment protocols are based on functional status and comorbidities in addition to tumor characteristics such as location, size, and histology.

Surgery is the primary treatment for resectable STS. In disease recurrence, the decision to operate is affected by tumor grade, histology, response to systemic therapy, and ability to obtain adequate margins if resected [13]. The status of the surgical margins is the most important surgical variable that influences local control [14,15,16,17]. Limb salvage is preferred to amputations when possible [16,17,18,19,20,21,22]. Amputation is generally reserved for patients with severe comorbidities, predisposing them to wound complications and locally advanced or recurrent STS not amenable to resection [15].

Radiation therapy (RT) is beneficial in improving local control and reducing the risk of recurrence in addition to assisting with better functional outcomes. For patients with extremity sarcomas >5 cm, the addition of RT improves local control and has significant impact on limb salvage. Acute and late complications secondary to radiation can influence timing of RT [19]. Pre-operative RT can help improve surgical clear margins, allow for lower treatment dose, and improve functional outcomes, though it is associated with a higher risk of post-operative wound complications [19,20]. Post-operative RT allows for better determination of tumor characteristics, though a disadvantage, once again, is the need for higher treatment dose and related toxicities [19,20,21]. The current preferred dose for RT is 5000 cGy for pre-operative RT, while 6000–6600 cGy is generally used for post-operative RT. In some cases, a post-operative or intra-operative boost is delivered [19].

Neoadjuvant and/or adjuvant chemotherapy as a component of multimodal treatment in STS is beneficial to select high-risk patients to reduce the risk of local recurrence [22]. Doxorubicin (Adriamycin) and ifosfamide are the most effective drugs for sarcoma, and when combined with mesna make up the AIM chemotherapy regimen, which is the most common regimen used. Systemic therapy is the primary treatment for palliation in incurable metastatic sarcomas.

## 3. Assessment of the Sarcoma Patient

A rehabilitation consultation (physiatrist and or a physical therapist) is beneficial in the pre-habilitation and post-operative phase of treatment and should be offered to patients. A comprehensive physical assessment is critical for identifying and managing physical impairments commonly seen in sarcoma survivors [23,24]. This process begins with a thorough medical chart review, detailed history, and continues with a targeted musculoskeletal and neurological physical evaluation [23,24].

### 3.1. Medical History and Physical Examination

The initial assessment should encompass a thorough history, focusing on the type, location, and treatment of the sarcoma [23,24]. Critical elements include the patient’s treatment history (surgical interventions, radiation therapy, chemotherapy), post-operative complications, and the presence of chronic pain or other symptoms [23,24]. A thorough chart review is indispensable for understanding the extent of surgical resection, the specific muscles and nerves involved, and any radiation-induced complications [19,20,21,22,23]. For instance, understanding the radiation fields and doses are crucial for anticipating areas of fibrosis or nerve damage. Chart review should also include review of the patient’s co-morbid conditions such as presence of pulmonary metastasis, chronic obstructive pulmonary disease (COPD), diabetes mellitus and cardiovascular disease that could all have an impact on their post-sarcoma treatment recovery and rehabilitation program. It is also valuable to review pertinent imaging studies such as PET/CT scans, MRIs and X-rays whenever available to fully understand the extent of the disease and correlation with affected anatomy and physical function.

### 3.2. Musculoskeletal Examination

The musculoskeletal examination should focus on assessing muscle strength, joint range of motion, and signs of joint instability [23,24]. For patients with upper extremity sarcomas, a detailed shoulder examination is essential [23]. This includes evaluating rotator cuff integrity, scapular dyskinesis, and glenohumeral joint stability [23]. In lower extremity sarcomas, the examination should include assessments of hip and knee range of motion, strength, gait analysis, and balance testing [23,24]. Specific tests, such as the Trendelenburg sign, can help identify gluteal muscle weakness, while manual muscle testing can quantify deficits in specific muscle groups [23,24].

### 3.3. Neurological Examination

Given the potential for peripheral nerve involvement—either from the tumor or because of treatment—a thorough neurological examination is crucial [23]. This includes testing for sensory deficits, motor weakness, and reflex abnormalities [24]. In cases where peripheral neuropathy or isolated peripheral nerve injury is suspected, electromyography and nerve conduction studies may be indicated [24]. For example, assessing the integrity of the sciatic nerve in patients with pelvic sarcomas is essential, as it is commonly affected by both the tumor and subsequent treatments [23,24].

## 4. Physical Impairments in Sarcoma Survivors

Sarcoma survivors commonly experience a spectrum of physical impairments, which can affect their quality of life [25]. These impairments are heavily influenced by the tumor’s type, location, and the treatments received [25,26].

### 4.1. Shoulder Dysfunction

Sarcomas involving the upper extremities or shoulder girdle can lead to considerable functional limitations post-treatment [23]. Surgical resections often result in muscle weakness, reduced range of motion, and chronic pain, leading to shoulder dysfunction [23]. Radiation therapy can exacerbate these issues through fibrosis, causing joint stiffness and soft tissue contracture [13,23,27]. For instance, resection of the rotator cuff muscles or deltoid may result in significant loss of shoulder function, impacting activities of daily living (ADLs) and overall independence [23,24].

### 4.2. Lower Back and Hip Muscle Weakness

Sarcomas of the pelvis or lower extremities often result in substantial weakness in the lower back and hip muscles [23,25]. Such weakness contributes to gait abnormalities and balance difficulties [24]. For example, a patient with a pelvic sarcoma requiring extensive resection may develop gluteal muscle weakness, leading to a Trendelenburg gait, characterized by a drop of the pelvis on the contralateral side during the stance phase [24,27]. Radiation-induced damage to muscles and nerves can further impair function, contributing to chronic pain and significant disability [13,23].

### 4.3. Regional Impairments

The specific location of the sarcoma significantly influences the pattern of physical impairments [23,26]. For example, a sarcoma in the thigh treated with limb-sparing surgery may leave the patient with residual muscle weakness, joint instability, or peripheral neuropathy, all of which impede mobility [23,25,26]. Similarly, radiation therapy can cause localized tissue damage, leading to fibrosis, lymphedema, and compromised circulation, which may worsen functional outcomes [19,23,24].

### 4.4. Gait and Balance Difficulties

Gait disturbances are prevalent among sarcoma survivors, particularly those with tumors in the lower extremities or pelvis [23]. Contributing factors include muscle weakness, joint stiffness, and proprioceptive deficits [23,27]. Balance issues may arise from a combination of these factors, increasing the risk of falls and subsequent injury [23,27]. For example, patients with sarcomas in the lower limb might exhibit a reduced ability to perform single-leg stance tasks, reflecting both muscular and proprioceptive deficits [23,27].

The presence of the physical impairments mentioned above can contribute to the development of activity limitations and participation restrictions, which can have an adverse impact on the person’s ability to achieve their educational and professional aspirations, develop and maintain social connections, engage in healthy activities such as exercise and in other activities that bring meaning and fulfillment to their lives.

## 5. Rehabilitation Continuum and Team Members in the Care of Soft Tissue Sarcoma Survivors

Navigating the journey of recovery from STS involves more than just surgical and/or medical treatments; it requires a robust rehabilitation strategy powered by a dedicated team of professionals. A multidisciplinary rehabilitation plan can help minimize symptoms and sequelae which negatively affect the patient’s function and quality of life [24] including pain, chemotherapy-induced peripheral neuropathy, radiation fibrosis, activity restrictions following surgical excision, amputation, bowel and bladder dysfunction, and lymphedema [28]. The rehabilitation team members—cancer physiatrists, physical therapists, occupational therapists, speech language pathologists, rehabilitation oncology nurses, neuropsychologists, etc.—each play crucial roles in tailoring an individualized delivery system that addresses both the physical and emotional needs of patients with STS. Rehabilitation can be carried out in different settings: acute inpatient rehabilitation (IPR), skilled nursing facilities (SNF)/long-term care facilities (LTAC), home health rehabilitation services, and outpatient/clinic-based rehabilitation.

During acute hospitalization for cancer-directed treatment such as surgical resection of the sarcoma and/or chemoradiation, a patient’s function and need for recovery may necessitate admission to an inpatient rehabilitation program. To qualify for inpatient rehabilitation, patients will need to require ongoing medical management (i.e., post-operative pain control, hemodynamic monitoring/blood pressure, management of pre-morbid conditions, etc.) as well requiring a comprehensive rehabilitation program that includes physical therapy, occupational therapy, and/or speech language pathology. In addition, the patient must be able to participate and tolerate therapies at least 3 h a day and 5 days a week. Inpatient rehabilitation serves as a pivotal first step, allowing individuals to regain strength and mobility in a controlled environment where patients can receive constant support. For example, physical therapists focus on implementing different exercise modalities including aerobic and resistance training designed to restore function by promoting greater exercise tolerance and endurance, while occupational therapists work on helping patients adapt their daily activities including dressing, bathing, etc.

For patients unable to tolerate and participate with the exercise intensity required by acute inpatient rehabilitation, SNFs/LTACs may be a good alternative option, as they provide the medical supervision but with less rehabilitation therapy sessions. In some instances, functional recovery gained from IPR may not be sufficient for a safe home discharge and/or patients do not have enough support at home (no caregivers, etc.), and may warrant continuation for rehabilitation therapy in a less intense setting at SNF. LTAC facilities also have certain options like acute level of care such as ventilation management/weaning of patients, etc., but rehabilitation therapies are far less frequent due to medical limitations of the patients like those attached to a ventilator [29].

As patients transition to home health services, the emphasis shifts toward independence and adapting to life post-treatment. Home health rehabilitation therapists continue to guide recovery through personalized care plans that promote self-sufficiency while ensuring safety at home. The home program is ideal for patients as they continue to recover without the need of specialized equipment or for those patients with some physical limitations preventing them from transitioning to the outpatient rehabilitation immediately. The priority of home therapies is to maximize and ensure safety as well as physical function in the home environment with goal of transitioning to outpatient therapy [29].

Outpatient rehabilitation then becomes an essential component for many STS survivors, as it offers ongoing support and progress tracking through regular sessions that build upon previous gains. The outpatient program provides continuation of rehabilitation therapies and focuses on activities that require supervision using specific modalities and utilization of specialized equipment [29]. Therapies, whether physical therapy, occupational therapy, and/or speech therapy, continue to focus on specific impairments from the tumors, cancer-directed treatment complications/side effects, and/or generalized conditioning. This comprehensive approach not only enhances physical well-being but also fosters psychological resilience, empowering individuals to reclaim their lives after cancer treatment. The outpatient rehabilitation therapy typically comprises 2–3 visits per week for a 6–8-week duration with the plan to transition to an independent home exercise program. Goals include an effective exercise program through aerobic and/or resistance training with a focus on safety with minimal guidance. The patient’s cancer physiatrists’ rehabilitation and exercise prescription should include diagnosis, specific impairment, frequency, duration, and precautions to ensure formal and safe program. The collaborative efforts of the rehabilitation team create a network of care that inspires hope and facilitates healing during this challenging time.

## 6. Treatment Plans and Outcomes

Physical and functional impairment in patients with STS depends on the consensus treatment plan. Currently, complete surgical resection is the primary treatment [30] and is the only curative treatment for most STS; however, in some situations when en bloc resection is not feasible, such as the spine, chemoradiation is used for treatment. En bloc resection has lowered the need for an amputation for primary therapy of sarcomas to less than 10% [31]. Physical and functional impairment post-operatively depends on the surgical plan and approach depending on the stage/grade and size of the tumor. Functional outcomes and quality of life are significantly worse after amputation or major nerve resections [32]. Ideally, the patient should be evaluated by a cancer physiatrist to determine baseline function prior to any cancer-directed treatment, which includes surgery, radiotherapy and/or systemic treatments, which can be associated with a lower health-related quality of life [33]. If an impairment is already present, it is critical to address any pre-morbid impairment through a cancer pre-habilitation program to prevent a functional decline. Additionally, even without any significant pre-morbid conditions, it is important to educate the patient on the potential impairments that can occur. In the setting of neoadjuvant chemotherapy prior to surgical resection, the patient will benefit from a cancer pre-habilitation program to increase the patient’s functional status, endurance/stamina, and strength to minimize any post-operative complications and morbidity.

In the acute post-operative setting, patients are at significant risk for wound complication (17%), especially in the setting of post-operative RT [34]. Although early mobility is encouraged to prevent debility, it is important to ensure safety and precautions are in place during the rehabilitation process. Getting the patient to mobilize early is important to prevent debility in post-surgical patients, but constant communication with the surgeon or other treatment teams for clearance/activity limitations is critical to ensure the patient’s surgical incisions are healing appropriately to avoid any wound dehiscence and/or infections.

Post-operative complications induced functional impairment [35] can profoundly impact daily life, making it essential for rehabilitation strategies to prioritize gradual re-introduction of activities that promote participation in personal interests and social engagements. Evaluation of patients’ functional status and quality of life is critical to improve a patient’s functional outcome.

Patients undergoing amputation have greater functional impairments than those receiving conservative or limb-sparing surgery [36]. Reduced post-operative function correlates with poorer quality of life [37].

For patients undergoing limb salvage procedures nerve resection during surgery is associated with worse outcomes [38]. In addition, female gender (*p* = 0.03), lower extremity tumors (*p* < 0.01), and radiotherapy (RT, *p* = 0.02) are linked to lower Toronto Extremity Salvage Scores (TESS) [37].

A recent study by Fukushima et al. looked at the validity of subjective and objective measures to evaluate patients undergoing surgery for STS in the thigh. The Timed Up and Go Test (TUGT) is effective for evaluating walking speed, strength, and balance post-surgery and the Musculoskeletal Tumor Society (MSTS) score provides a subjective measure of limb function [39].

It would be beneficial to utilize tools like TUGT and MSTS for comprehensive functional evaluation and outcomes. Quadriceps and tibialis anterior muscle resections significantly affect post-operative physical function as reflected in TUGT scores [39]; thus, rehabilitation plans need to be tailored to mitigate deficits caused by specific muscle resections. Recommendations from this group included early rehabilitation and orthotic support for knee extension and ankle dorsiflexion [40].

A retrospective study by Tanaka et al. looked at knee flexion strength and post-operative function after knee flexor muscle resection for STS of the lower limbs [41]. The group found knee flexion strength decreased significantly with resection of flexor muscles, especially bilateral hamstrings (median strength drop to 27.3%) [41], and that reduced strength was associated with lower functional outcomes. Pre-habilitation and/or early post-operative rehabilitation is crucial in these patients.

A 6% fracture rate was observed in patients undergoing limb-sparing surgery and post-operative RT or chemotherapy [42]. Periosteal stripping has been shown to have a fracture risk, with a 5-year actuarial fracture rate of 8.6% [43]. Rehabilitation protocols should incorporate precautions, especially in patients with periosteal stripping, to minimize fracture risk [43].

In summary, for postoperative limb salvage patients, recommendations from prior studies include incorporating early and intensive individualized rehabilitation focusing on strength, balance, and functional recovery, monitor weight-bearing capacity and fracture risk, particularly in patients undergoing periosteal stripping or adjuvant therapies.

Patients facing limb amputation can benefit from an early assessment by a cancer physiatrist to prepare them for the surgery. Post-operatively, it is essential for them to undergo an evaluation with a prosthetist to initiate pre-prosthetic training immediately. This approach is the most effective way to help patients recover and regain their independence as quickly and thoroughly as possible. In some patients with high-grade STS, a newer surgical approach involving prosthetic replacement reconstruction after lower extremity tumor resection is emerging; however, major complications include infection and aseptic loosening, and the 5-year prosthetic survival rate was at 51.1% [44]. Despite the ongoing advancements in surgical techniques and technology, both pre-operative and post-operative rehabilitation will benefit patients significantly.

RT is used for treatment of STS after surgical procedure to decrease risk of local relapse. Careful planning to ensure coverage of the tumor margins and drain sites is important to benefit from the RT while avoiding irradiation of a large proportion of the cross-sectional area of the extremity so that lymphedema becomes a significant issue [45]. Another complication of RT is damage to the nearby healthy tissues, such as skin, fascia, muscles, nerves, and bones. This damage may result in pathological fractures and soft tissue fibrosis, further contributing to functional impairment on top of any loss of function that occurs after surgery.

It is essential for patients experiencing lymphedema due to surgery or radiation to be referred promptly to a cancer physiatrist. An assessment is crucial not only for preserving functionality but also for preventing the condition from deteriorating. Lymphedema therapists assist patients through techniques such as manual lymphatic drainage, wrapping, and compression therapy. Regular follow-up appointments are necessary to monitor adherence to the treatment plan and to check for potential infections like cellulitis.

Incorporating systemic therapy during the neoadjuvant or adjuvant treatment reduces the likelihood of recurrence and improves survival rates. However, the side effects and complications associated with these treatments may result in diminished functionality and a lower quality of life for patients. Depending on the type of chemotherapy used, systemic side effects can include immunosuppression, fatigue, overall weakness, and neuromuscular issues, among others, which may contribute to reduced exercise capacity. Although more research is needed, emerging evidence suggests that physical activity improves symptom management and quality of life for people undergoing chemotherapy for advanced cancers [46].

## 7. Rehabilitation Outcomes for Survivors of Soft Tissue Sarcomas

Tumor size, nerve, and bone resection as well as treatment-related complications affect functional outcomes in STS [36]. Outcome measures typically used include MSTS, Toronto Extremity Salvage Score (TESS), Timed Up and Go (TUG) test and the Disabilities of Arm, Shoulder, and Hand (DASH) or QuickDASH.

Functional outcomes have been researched in patients with STS with limb salvage procedures as well as amputation. Studies in patients post-internal and external hemipelvectomy included functional outcomes data for both bone and STS [47,48,49,50]. Studies have demonstrated that loss of knee extensor strength with femoral nerve resection [51], or quadriceps muscle resection [39,52,53], result in impairments in gait. Early mobilization and therapy in the post-operative setting have been demonstrated to have improved functional outcomes [54] and reduced hospital length of stay and readmission. Need for reconstructive surgery, higher body mass index and older patients are more likely to have lower functional outcomes in patients with lower extremity limb salvage procedures [55]. For upper extremity sarcomas, amputation and major nerve resection had the most impact on functional outcomes [32]. In both upper extremity and lower extremity STS, limb salvage has superior functional outcomes [56,57]. Though, return to sports-related activities is possible after limb salvage procedures with appropriate rehabilitation [58].

## 8. Future Directions

Studies evaluating prehabilitation outcomes as well as standardized protocols for post-treatment rehabilitation in STS patients will help with improving rehabilitation plans in these patients. Challenges in prospective trials are the wide variations in sarcoma treatment and the low incidence of these tumors which could be overcome with more multi-institutional studies after initial pilot studies. There is a current study being conducted for individualized prehabilitation in lower extremity soft tissue sarcomas which can help inform future studies at other sites of soft tissue sarcoma [59]^.^ We can also harness the electronic health record data in these patients to identify patients who may benefit most from prehabilitation and post-treatment rehabilitation. This will also help with more individualized rehabilitation treatment plans. Studies that focus on return to work as well as avocational activities are also lacking in this patient population and would be very useful in improving the quality of life for this population.

## 9. Conclusions

Sarcomas and their treatment with surgery, chemotherapy and radiation therapy can contribute to the development of significant physical impairments that can have a profound adverse impact on survivors’ physical function and quality of life. It is recommended that patients diagnosed with sarcomas be referred to physiatrists prior to start or early during cancer treatment. Through interventions such as pre-rehabilitation as well as a pro-active surveillance and treatment approach to common physical impairments involving the affected areas, rehabilitation team members can improve the physical function and quality of life of sarcoma survivors.

## Data Availability

Not applicable.
